# Study on Oxygen Evolution Reaction Performance of Jarosite/C Composites

**DOI:** 10.3390/ma15020668

**Published:** 2022-01-17

**Authors:** Junxue Chen, Sijia Li, Zizheng Qu, Zhonglin Li, Ding Wang, Jialong Shen, Yibing Li

**Affiliations:** Department of Materials Science and Engineering, Guilin University of Technology, Guilin 541000, China; cchenjunxue@gmail.com (J.C.); lisj701023@gmail.com (S.L.); dadpro01@gmail.com (Z.Q.); dahe121133@gmail.com (Z.L.); dingnvhuang@gmail.com (D.W.)

**Keywords:** jarosite, ammoniojarosite, electrocatalyst, oxygen evolution reaction, stability

## Abstract

In the electrolysis of water process, hydrogen is produced and the anodic oxygen evolution reaction (OER) dominates the reaction rate of the entire process. Currently, OER catalysts mostly consist of noble metal (NM) catalysts, which cannot be applied in industries due to the high price. It is of great importance to developing low-cost catalysts materials as NM materials substitution. In this work, jarosite (AFe_3_(SO_4_)_2_(OH)_6_, A = K^+^, Na^+^, NH^4+^, H_3_O^+^) was synthesized by a one-step method, and its OER catalytic performance was studied using catalytic slurry (the weight ratios of jarosite and conductive carbon black are 2:1, 1:1 and 1:2). Microstructures and functional groups of synthesized material were analyzed using XRD, SEM, FI-IR, etc. The OER catalytic performance of (NH_4_)Fe_3_(SO_4_)_2_(OH)_6_/conductive carbon black were examined by LSV, Tafel, EIS, ECSA, etc. The study found that the OER has the best catalytic performance when the weight ratio of (NH_4_)Fe_3_(SO_4_)_2_(OH)_6_ to conductive carbon black is 2:1. It requires only 376 mV overpotential to generate current densities of 10 mA cm^−2^ with a small Tafel slope (82.42 mV dec^−1^) and large C_dl_ value (26.17 mF cm^−2^).

## 1. Introduction

With the increasingly negative impact of fossil fuels on the environment, it is with a huge demand that modern science and technology need to pursue clean and sustainable energy [1,2]. Hydrogen production by water splitting, as a technology for producing clean energy, has caused extensive researches [3,4]. The electrochemical water splitting process includes the oxygen evolution reaction (OER) at the anode and the hydrogen evolution reaction (HER) at the cathode. The HER reaction is a two-electron transfer process, while OER is a four-electron transfer process, whose higher energy barrier dominates the rate of the cathodic hydrogen production [5]. Therefore, the development and research of cost-effective OER catalysts with high activity and long-periodic cycle stability is a primary task.

Noble-metal-based materials, including IrO_2_ and RuO_2_, are state-of-the-art OER electrocatalysts because of their high electrocatalytic OER activity both in alkaline and acidic solutions [6,7,8]. However, the large-scale application of IrO_2_ and RuO_2_ in OER is severely limited not only by the high cost but also by the scarcity of Ir and Ru [9]. Thus far, considerable research efforts have been devoted to the exploration of low-cost and highly active noble-metal-free catalysts to replace expensive and scarce precious catalysts [10,11,12]. Especially for transition metal Fe-based materials, including oxides/hydroxides [13,14,15], chalcogenides [16], phosphides [16,17], and nitrides [18], which have been investigated extensively as promising candidates for the OER.

Compared with these compounds, Fe-based polyanionic compounds [1,2,3,4], such as jarosite, are an earth-abundant natural mineral that belongs to the alunite supergroup with the formula AFe_3_(SO_4_)_2_(OH)_6_, where A represents different monovalent cations, such as K^+^, Na^+^, NH_4_^+^, and H_3_O^+^. At present, jarosite has been extensively studied by the acid leach mining industry due to the precipitation of jarosite in acidic media—a crucial step that allows for the physical separation of Fe^3+^ and other cations from the leach solution [19,20,21,22,23]. As a result, these refining plants produce large amounts of environmentally hazardous jarosite wastes that currently provide no commercial value. Fortunately, several researchers have explored the use of jarosite as a cathode in the lithium and sodium-ion battery [24,25,26]. According to our best efforts, there is no research on jarosite in OER so far. Therefore, exploring and tapping the potential of these environmentally harmful jarosite wastes in the field of OER is an attractive strategy to provide economic advantages for jarosite.

In this work, the design of applying jarosite to OER catalytic material is proposed. A simple one-step method was used to synthesize four various types of jarosite materials ((NaFe_3_(SO_4_)_2_(OH)_6_), KFe_3_(SO_4_)_2_(OH)_6_), (NH_4_)Fe_3_(SO_4_)_2_(OH)_6_, and ((H_3_O)Fe_3_(SO_4_)_2_(OH)_6_)). We explored the OER performance of these four catalysts under acidic, neutral and alkaline conditions. The electrochemical test results suggest that (NH_4_)Fe_3_(SO_4_)_2_(OH)_6_ shows the best OER activity among the four catalysts. When the weight ratio of (NH_4_)Fe_3_(SO_4_)_2_(OH)_6_ to conductive carbon black is 2:1, the overpotential of (NH_4_)Fe_3_(SO_4_)_2_(OH)_6_ is 376 mV at a current density of 10 mA cm^−2^ in alkaline conditions. Although this performance is not comparable to that of precious metals (IrO_2_), there is a lot of room to improve the performance, which is our future research task. In short, exploring the application of jarosite waste in OER can not only protect the environment but also improve its economic value.

## 2. Experimental Section

### 2.1. Materials

Fe(SO_4_)∙7H_2_O (99.0~101.0%), K_2_CO_3_ (99%), Na_2_CO_3_ (99.8%), (NH_4_)_2_SO_4_ (98.0%), NH_3_∙7H_2_O (25.0~28.0%) and C_2_H_5_OH (99.7%) were purchased from Guangxi Dragon Technology Company (Guangxi, China). Conductive carbon black (Ketjenblack) and Nafion solution (5 wt%) were purchased from Suzhou Yilongsheng Energy Technology Co., Ltd. (Suzhou, China) All chemicals used were of analytical grade and there was no need to further purification.

### 2.2. Preparation of (A)Fe_3_(SO_4_)_2_(OH)_6_, (A = K^+^, Na^+^ and NH_4_^+^)

FeSO_4_ and (NH_4_)_2_SO_4_ were dissolved in 250 mL and 100 mL of deionized water to form solution A (0.02 M FeSO_4_) and solution B (0.5 M (NH_4_)_2_SO_4_), respectively. Dilute H_2_O_2_ solution was added dropwise to solution A to oxidize Fe^2+^ to Fe^3+^ and then solution B was injected into the above solution with a water bath at a constant temperature of 95 °C and stirred magnetically for 3 h, meanwhile, the pH value of the mixed solution was maintained throughout at 1.5–2.0 with 1 M ammonia solution. Finally, the yellow precipitate (NH_4_)Fe_3_(SO_4_)_2_(OH)_6_ catalyst was collected, washed with deionized water and then dried in a vacuum at 80 °C. The main synthesis procedure for Na/KFe_3_(SO_4_)_2_(OH)_6_ is similar to that for (NH_4_)Fe_3_(SO_4_)_2_(OH)_6_, corresponding to the use of 0.5 M Na_2_CO_3_ and K_2_CO_3_ instead of 0.5 M (NH_4_)_2_SO_4_ solution and the replacement of the pH adjuster with 1 M Na_2_CO_3_ and K_2_CO_3_ solution, respectively.

### 2.3. Preparation of (H_2_O)Fe_3_(SO_4_)_2_(OH)_6_

A certain amount of FeSO_4_ was dispersed in 70 mL of deionized water, with stirring at 95 °C. H_2_O_2_ was used to oxidize Fe^2+^ to Fe^3+^. After it was completely oxidized, the reactants were transferred to a 100 mL reactor at 120 °C and kept for 12 h. The final product was collected after filtration and washed with deionized water several times.

### 2.4. Preparation of Working Electrode

Add 10 mg NH_4_-Fe_3_@KB-1((NH_4_)Fe_3_(SO_4_)_2_(OH)_6_ and conductive carbon black with a mass ratio of 2:1). These were dispersed into a mixed solvent of Nafion (30 µL), anhydrous ethanol (400 µL) and deionized water (600 µL). Form a uniform dispersion after ultrasonic for 30 min, then use a pipette gun to take the dispersion (4 µL), add to the glassy carbon electrode and dry. The working electrodes of other catalytic materials are prepared by using the same method.

### 2.5. Characterization

Scanning electron microscopy (SEM, Hitachi Works, Ltd., Tokyo, Japan) was conducted on S-4800 at 5 kV to perform microstructure analysis. The phase structure of the sample was analyzed by a X-ray powder diffractometer (XRD, X’Pert PRO, PANalytical B.V, Cu Kα, 40 kV, 40 mA, λ = 1.54056 Å, PANalytical B.V., Almelo, The Netherlands) at a scanning rate of 5°∙min^−1^. The functional groups of the samples were analyzed by Fourier transform infrared (FTIR, Thermo Nexus 407 spectrometer, White Bear Lake, MN, USA) and the laser Raman confocal microscope Raman spectrometer (Raman, Thermo Fisher Scientific DXR, thermoelectric company, 532 nm, White Bear Lake, MN, USA). Transmission electron microscopy (TEM, JEOL, Beijing, China) images were collected on Titan G260-300 at an acceleration voltage of 200 kV. Before BET and BJH measured via Surface area and pore porosimetry analyzer NoVA 1200e (Quantachrome Instruments, Shanghai, China), all samples were degassed for 5 h at 100 °C.

### 2.6. Electrochemical Measurements

All electrochemical measurements were conducted on a computer-controlled CHI 760E electrochemical workstation with a conventional three-electrode system. The glassy carbon electrode with a diameter of 3 mm was used as a working electrode, Ag/AgCl electrode and Hg/HgO electrode were respectively used as the reference electrode for the acidic (neutral) system and alkaline system, and graphite rods were used as a counter electrode. The measurements were performed in three different electrolytes, 0.05 M H_2_SO_4_, 1 M KOH, and 1 M PBS. All the potentials were converted to reversible hydrogen electrode (RHE) based on the formula E_RHE_ = E_Hg/HgO_ + 0.0591 × pH + 0.098 and E_RHE_ = E_Ag/AgCl_ + 0.0591 × pH + 0.1976. The polarization curves were measured at 5 mV s^−1^ and iR-corrected. Tafel plots were calculated using the Tafel formula *η* = *b* log *j* + *a*, where *j* is the current density, *b* is the Tafel slope, and *a* is the intercept relative to the exchange current density. EIS measurements were conducted under a particular applied potential in the frequency range 0.1 Hz to 100 kHz. The electrochemically active surface area (ECSA) was estimated by the double-layer capacitance (C_dl_). The time-current curve was measured at a fixed voltage corresponding to 10 mA cm^−2^ of current density. All tests were performed at room temperature.

## 3. Results and Discussion

### 3.1. Characterization of Samples

**Figure 1:**
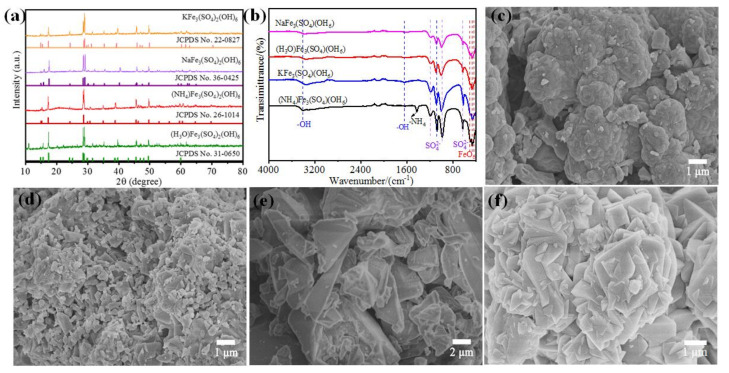
(**a**) XRD patterns and (**b**) FT-IR spectra of various types of jarosite. SEM images of (**c**) KFe_3_(SO_4_)_2_(OH)_6_, (**d**) NaFe_3_(SO_4_)_2_(OH)_6_, (**e**) (H_3_O)Fe_3_(SO_4_)_2_(OH)_6_ and (**f**) (NH_4_)Fe_3_(SO_4_)_2_(OH)_6_.

Four various types of jarosite were synthesized, named NaFe_3_(SO_4_)_2_(OH), KFe_3_(SO4)_2_(OH)_6_, (NH_4_)Fe_3_(SO_4_)_2_(OH)_6_ and (H_3_O)Fe_3_(SO_4_)_2_(OH)_6_, respectively. The crystal structure of various types of jarosite was firstly investigated by X-ray diffraction (XRD). As shown in Figure 1a, the diffraction pattern of the as-prepared jarosite can be indexed to the hexagonal system with a space group of R3m, suggesting the successful preparation of the jarosite samples.

Figure 1b shows the infrared spectrum test chart of the jarosite. The infrared absorption peaks of different jarosite appear at similar positions. The peaks appearing at 469 cm^−1^ and 502 cm^−1^ are Fe-O peaks. The corresponding peaks at 624 cm^−1^, 1082 cm^−1^, and 1204 cm^−1^ are SO_4_^2−^. The broad and strong absorption peaks at 1004 cm^−1^ and 3416~3700 cm^−1^ are the stretching vibrations of –OH and the weaker absorption peak at 1638 cm^−1^ is caused by the bending vibration of H_2_O [27,28]. A sharp peak appears at 1425 cm^−1^ in the infrared spectrum of (NH_4_)Fe_3_(SO_4_)_2_(OH)_6_, which is regarded as the absorption of the –NH_4_ peak. According to the findings in the report [15], transition metal hydroxides have good OER catalytic performance and the presence of hydroxyl groups in jarosite makes it possible to have OER catalytic performance. This view is confirmed in the following electrochemical performance test.

It can be seen from the figure that KFe_3_(SO_4_)_2_(OH)_6_ (Figure 1c) and NaFe_3_(SO_4_)_2_(OH)_6_ (Figure 1d) have similar morphologies. Both of them are densely packed. The precipitation rate of KFe_3_(SO_4_)_2_(OH)_6_ is fast and the sample morphology has not yet been completely formed before it settles together. In the morphology of NaFe_3_(SO_4_)_2_(OH)_6_, it can be observed that they are stacked together in a rhombic structure, which has not yet been completely formed. It can be seen from Figure 1e that the particle diameter is larger and the shape is irregular. (NH_4_)Fe_3_(SO_4_)_2_(OH)_6_ (Figure 1f) is uniformly distributed in lumps of different sizes while particles do not appear to pile up, showing a larger specific surface area.

**Figure 2:**
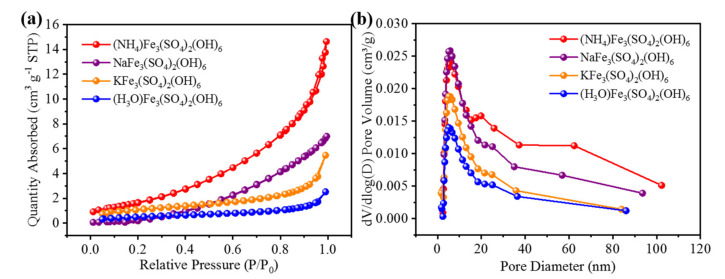
(**a**) N_2_ adsorption-desorption isotherm curves, (**b**) the corresponding pore size distribution curves.

The nitrogen adsorption-desorption isotherm curves of the as-synthesized catalyst under various pressures were characterized with a Surface Area and Pore Porosimetry Analyzer NoVA 1200e., and the specific surface area and pore size distribution were calculated via Brumaire-Emmett-Teller(BET) and Barret-Joyner-Hallender (BJH) methods. As shown in Figure 2, (NH_4_)Fe_3_(SO_4_)_2_(OH)_6_ displays the highest BET surface areas of 6.5845 m^2^ g^−1^, which is higher than that of KFe_3_(SO_4_)_2_(OH)_6_ (4.6879 m^2^ g^−1^), NaFe_3_(SO_4_)_2_(OH)_6_ (4.1587 m^2^ g^−1^), and (H_3_O)Fe_3_(SO_4_)_2_(OH)_6_ (2.5179 m^2^ g^−1^). The pore size distribution curves of the four materials in Figure 2b suggest the existence of a mesoporous structure (~8 nm). The large specific surface area of the catalyst is very beneficial for the exposure of catalytic active sites for OER.

**Figure 3:**
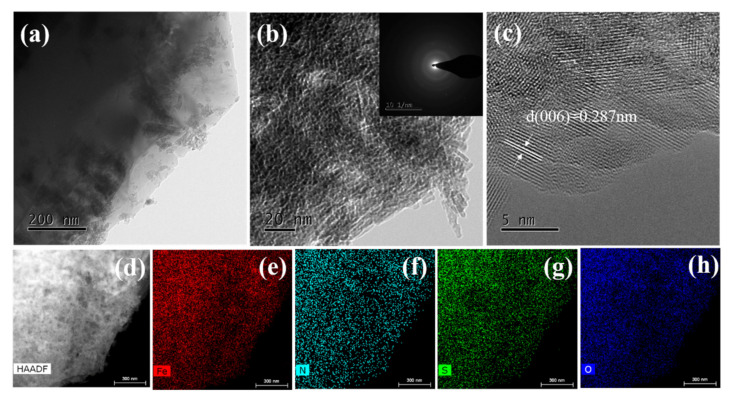
(**a**,**b**) TEM images (the inset shows SAED), (**c**) HRTEM image, and (**d**–**h**) HAADF-TEM diagrams of (NH_4_)Fe_3_(SO_4_)_2_(OH)_6_ and the corresponding EDS elemental mapping images.

Transmission electron microscopy (TEM) was carried out to further identify the details of samples. Figure 3a,b shows the lamellar structure of the sample. It shows some branch-like structures, which can provide more active sites. The selected-area electron-diffraction (SAED) pattern (inset of Figure 3b) of (NH_4_)Fe_3_(SO_4_)_2_(OH)_6_ was also recorded. It displays the weak diffraction rings, which further explained how the prepared (NH_4_)Fe_3_(SO_4_)_2_(OH)_6_ possesses poor crystallization form. Figure 3c shows the recorded high-resolution transmission electron microscopy (HRTEM) image of the (NH_4_)Fe_3_(SO_4_)_2_(OH)_6_. The interplanar spacing of 0.287 nm was indexed matching the (006) crystal plane of (NH_4_)Fe_3_(SO_4_)_2_(OH), which is in good agreement with the XRD spectra. Furthermore, the high-angle annular dark-field scanning-TEM (HAADF–STEM) and its corresponding mapping were employed to analyze the distribution of the elements in the (NH_4_)Fe_3_(SO_4_)_2_(OH)_6_ catalyst. It shows that Fe, N, O, and S are evenly distributed across the entire nanoparticles without any noticeable segregation.

### 3.2. Electrochemical Analysis

**Figure 4:**
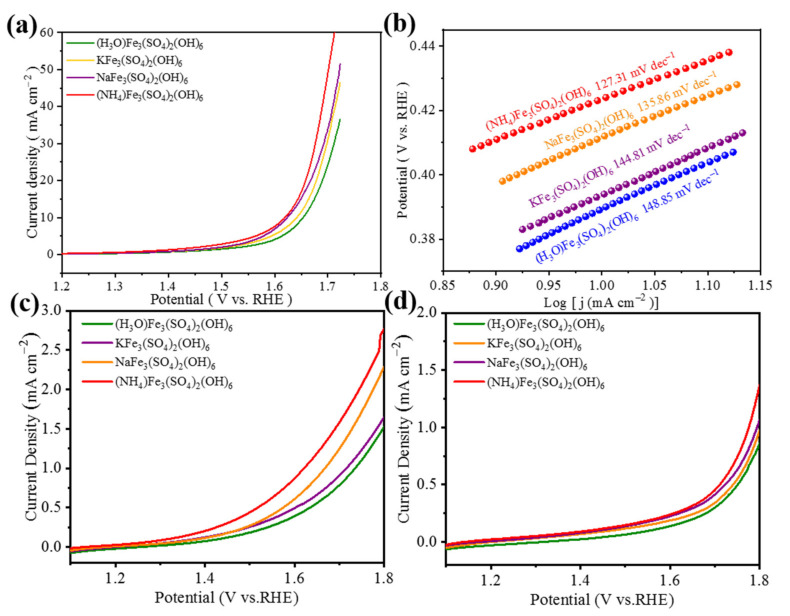
Polarization curves for jarosite to conductive carbon black ratio of 1:1, (**a**) 1 M KOH (pH = 14) polarization curves, (**b**) Tafel plots derived from the Ph = 14 polarization curves, (**c**) 0.05 M H_2_SO_4_ (pH = 1) polarization curves, (**d**) 1 M PBS (pH = 7) polarization curves.

To increase the electronic conductivity of the jarosite, the catalyst slurry with a weight ratio of 1:1 (jarosite to conductive carbon black) was prepared and an OER polarization curve performance test was conducted. As shown in Figure 4a, when the current density is 10 mA cm^−2^, the overpotentials of KFe_3_(SO_4_)_2_(OH)_6,_ NaFe_3_(SO_4_)_2_(OH)_6_, (H_3_O)Fe_3_(SO_4_)_2_(OH)_6,_ and(NH_4_)Fe_3_(SO_4_)_2_(OH)_6_ are 412 mV, 400 mV, 424 mV, and 394 mV, respectively. Meanwhile, the Tafel slope of the (NH_4_)Fe_3_(SO_4_)_2_(OH)_6_ is 127.31 mV dec^−1^, which is smaller than those of the NaFe_3_(SO_4_)_2_(OH)_6_ (135.86 mV dec^−1^), KFe_3_(SO_4_)_2_(OH)_6_ (144.81 mV dec^−1^) and (H_3_O)Fe_3_(SO_4_)_2_(OH)_6_ (148.85 mV dec^−1^), which indicates that (NH_4_)Fe_3_(SO_4_)_2_(OH)_6_ shows an excellent OER activity among the four jarosite catalysts.

**Figure 5:**
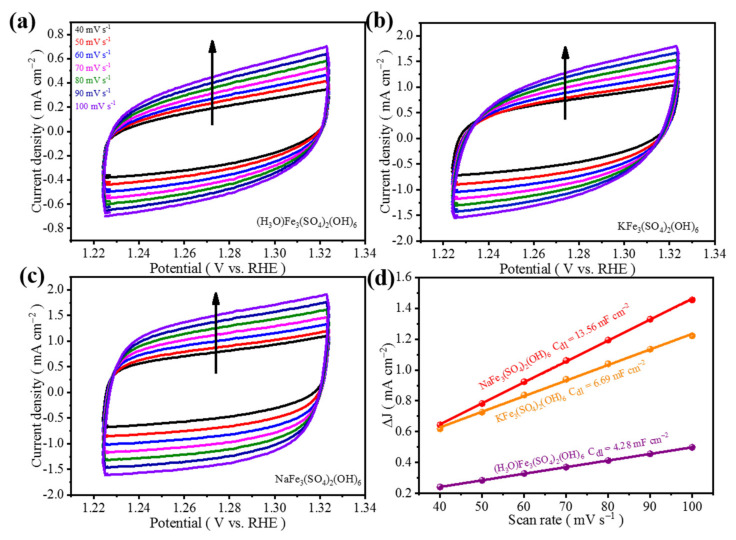
CV curves of (**a**–**c**) (H_3_O)Fe_3_(SO_4_)_2_(OH)_6_, KFe_3_(SO_4_)_2_(OH)_6_, and NaFe_3_(SO_4_)_2_(OH)_6_ at different scan rates, (**d**) C_dl_ diagram of (H_3_O)Fe_3_(SO_4_)_2_(OH)_6_, KFe_3_(SO_4_)_2_(OH)_6_, and NaFe_3_(SO_4_)_2_(OH)_6_.

In addition, OER tests were carried out on four catalyst materials in the acidic (pH = 1 H_2_SO_4_) and neutral (pH = 7 PBS) solution. As shown in Figure 5c,d, the catalytic performance of (NH_4_)Fe_3_(SO_4_)_2_(OH)_6_ in the acidic and neutral solution is better than the other three materials. However, the OER performance of (NH_4_)Fe_3_(SO_4_)_2_(OH)_6_ in the acidic and neutral condition is far inferior to that in the alkaline condition. Therefore, we will take alkaline conditions as an example to focus on the (NH_4_)Fe_3_(SO_4_)_2_(OH)_6_ catalyst.

**Figure 6:**
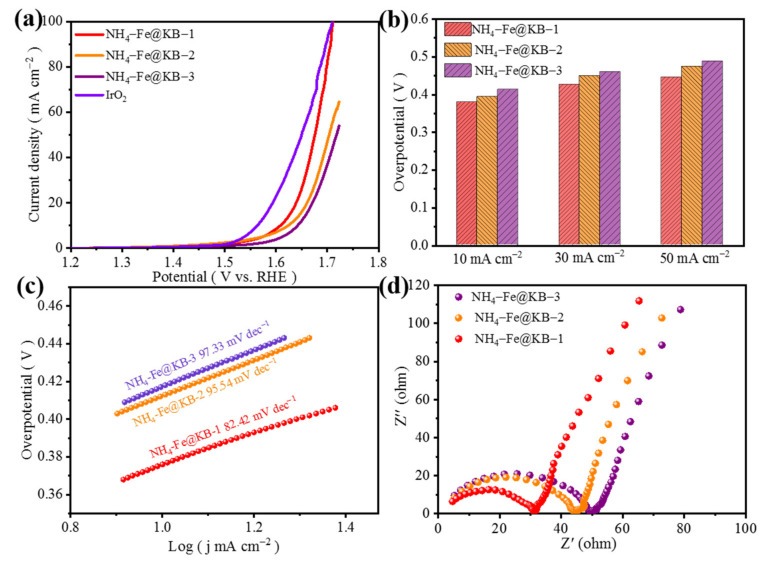
(**a**) NH_4_-Fe_3_@KB-1, NH_4_-Fe_3_@KB-2, NH_4_-Fe_3_@KB-3 and IrO_2_ polarization curves; (**b**) NH_4_-Fe_3_@KB-1, NH_4_-Fe_3_@KB-2, NH_4_-Fe_3_@KB-3 at overpotentials reaching current densities of 10, 50, and 100 mA cm^−2^; (**c**,**d**) the Tafel slope and EIS diagram of NH_4_-Fe_3_@KB-1, NH_4_-Fe_3_@KB-2, NH_4_-Fe_3_@KB-3.

The electrochemical double-layer capacitance (C_dl_) approach was applied to estimate the electrocatalytic active surface area (ECSA) from cyclic voltammetry curves at various scan rates over a small potential range. The (NH_4_)Fe_3_(SO_4_)_2_(OH)_6_ electrode possesses the largest C_dl_ of 15.49 mF cm^−2^ compared to those of KFe_3_(SO_4_)_2_(OH)_6_ (6.69 mF cm^−2^), NaFe_3_(SO_4_)_2_(OH)_6_ (13.56 mF cm^−2^), and (H_3_O)Fe_3_(SO_4_)_2_(OH)_6_ (4.28 mF cm^−2^) (Figure 5 and Figure 6), showing indeed that a larger ECSA of (NH_4_)Fe_3_(SO_4_)_2_(OH)_6_ allows for more exposed active sites to promote OER performance.

Different ratios of catalyst powder and conductive carbon black may affect the results. The different weight ratios of (NH_4_)Fe_3_(SO_4_)_2_(OH)_6_ and conductive carbon black (2:1, 1:1, 1:2) are prepared and the total mass of 10 mg is guaranteed. The samples are referred to as NH_4_-Fe_3_@KB-1, NH_4_-Fe@KB-2, NH_4_-Fe@KB-3 and IrO_2_. The OER polarization curve test was performed on them in 1 M KOH electrolyte saturated with oxygen, and the test results are shown in Figure 6a. Additionally, NH_4_-Fe_3_@KB-1 has better OER catalytic performance. When the current density is 10 mA cm^−2^, the overpotential of NH_4_-Fe_3_@KB-1 is 379 mV, and it is 15 mV and 34 mV lower than NH_4_-Fe_3_@KB-2 and NH_4_-Fe_3_@KB-3, respectively. Furthermore, (NH_4_)Fe_3_(SO_4_)_2_(OH)_6_ and IrO_2_ have the same overpotential when the current density is 100 mA cm^−2^. When the current density is 30 mA cm^−2^ and 50 mA cm^−2^, the overpotential of NH_4_-Fe_3_@KB-1 is still the lowest (Figure 6b). NH_4_-Fe_3_@KB-1 has a higher current density with the same measurement conditions.

To get insight into the OER kinetics, the Tafel slope values were calculated from the steady-state OER polarization curves. As shown in Figure 6c, NH_4_-Fe_3_@KB-1 (82.42 mV dec^−1^) has the smallest Tafel slope. Figure 6d is the AC impedance (EIS) test results of NH_4_-Fe_3_@KB-1, NH_4_-Fe_3_@KB-2 and NH_4_-Fe_3_@KB-3. The charge transfer resistance of NH_4_-Fe_3_@KB-1 is significantly smaller than that of NH_4_-Fe_3_@KB-2 and NH_4_-Fe_3_@KB-3, which suggests the catalytic interface and the electrolyte have a faster charge transfer rate.

**Figure 7:**
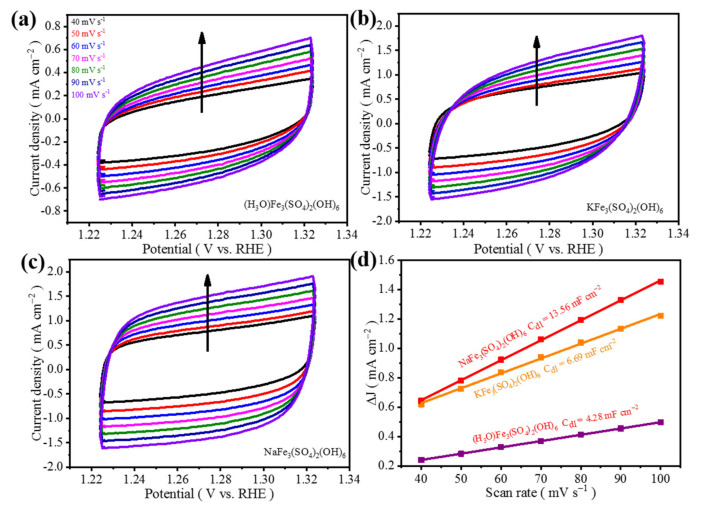
CV curves of (**a**) NH_4_-Fe_3_@KB-1, (**b**) NH_4_-Fe_3_@KB-2, and (**c**) NH_4_-Fe_3_@KB-3 at different scan rates; (**d**) C_dl_ diagram of NH_4_-Fe_3_@KB-1, NH_4_-Fe_3_@KB-2, and NH_4_-Fe_3_@KB-3.

The slope was calculated to get the C_dl_ value and the test result is shown in Figure 7. NH_4_-Fe_3_@KB-1 has the largest C_dl_ value of 26.17 mF cm^−2^, indicating that the ECSA of NH_4_-Fe_3_@KB-1 is large. This is allowing more active sites to be exposed and promotes the catalytic process of OER. This also explains the good OER catalytic performance of NH_4_-Fe_3_@KB-1.

**Figure 8:**
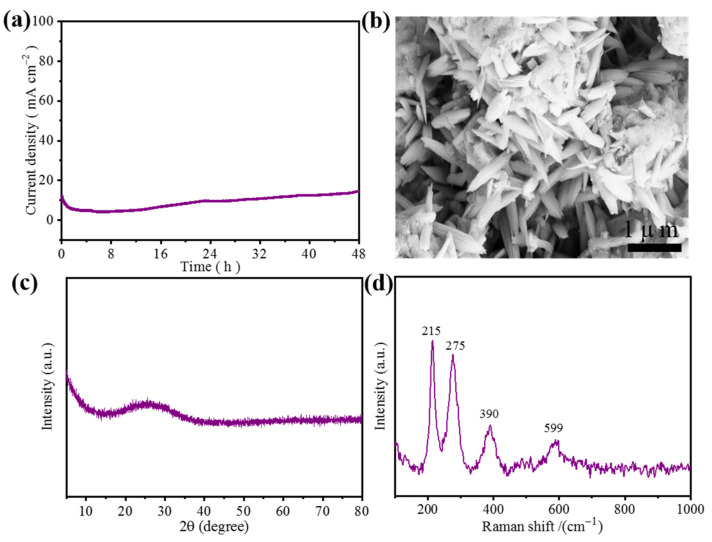
(**a**) The chronoamperometric curve for NH_4_-Fe_3_@KB-1, (**b**) SEM after stability test, (**c**) XRD after stability test, (**d**) Raman after stability test.

Additionally, durability was another significant parameter of the catalyst for OER. Through the i-*t* test, the stability of NH_4_-Fe_3_@KB-1 was evaluated, and the test was carried out for 48 h at a constant voltage of 0.68 V (vs. Hg/HgO) with a current density equal to 10 mA cm^−2^. The test results are shown in Figure 8a. With the increase in test time, the current density of NH_4_-Fe_3_@KB-1 increases slightly around 10 mA cm^−2^, which may be caused by the burst of oxygen bubbles generated during the test. Overall, NH_4_-Fe_3_@KB-1 still shows good stability. The structure and composition of (NH_4_)Fe_3_(SO_4_)_2_(OH)_6_ after the stability test was also studied in detail, with the SEM image of (NH_4_)Fe_3_(SO_4_)_2_(OH)_6_ (Figure 8b) after stability test displaying a newly formed rice-like structure, indicating that the (NH_4_)Fe_3_(SO_4_)_2_(OH)_6_ catalyst may have undergone surface reconstruction during electrolysis. In addition, the XRD pattern (Figure 8c) of the (NH_4_)Fe_3_(SO_4_)_2_(OH)_6_ catalyst showed an amorphous feature after OER. As with many reported works [29,30], the catalyst undergoes a surface reconstruction accompanied by the appearance of an amorphous structure (e.g., oxyhydroxide species) during the OER process, and the observed amorphous feature was further analyzed by Raman spectroscopy. As shown in Figure 8d, the four Raman bands at 215, 275, 390 and 599 cm^–1^ represent the phase of FeOOH, which is well-matched with the literature reports [31,32,33]. Therefore, it might be reasonable to conclude that (NH_4_)Fe_3_(SO_4_)_2_(OH)_6_ was transformed into amorphous FeOOH during the OER process.

## 4. Conclusions

In this work, a simple hydrothermal method was used to successfully prepare the jarosite. Furthermore, (NH_4_)Fe_3_(SO_4_)_2_(OH)_6_ shows the best catalytic performance. The OER catalytic performance of (NH_4_)Fe_3_(SO_4_)_2_(OH)_6_ and conductive carbon black with different weight ratios were further explored. The OER catalytic performance is best when the weight ratio of (NH_4_)Fe_3_(SO_4_)_2_(OH)_6_ to conductive carbon black is 2:1. Additionally, NH_4_-Fe_3_@KB-1 has a lower starting potential of 1.42 V (vs. RHE) and Tafel slope (82.42 mV dec^−1^). It also showed a small charge transfer resistance and a large C_dl_ (26.17 mF cm^−2^). The raw materials for preparing the synthetic are easily obtained and are low in price. Experimental results show that jarosite has a broad development space and further research is needed to improve its OER performance.

## Data Availability

The data used to support the findings of this study are available from the corresponding author upon request.
